# Conditions Associated With Hypertriglyceridemia in Adult Patients in a Tertiary Care Center in Basrah, Iraq

**DOI:** 10.7759/cureus.67609

**Published:** 2024-08-23

**Authors:** Issa Altemeemy, Nassar T Alibrahim, Qusay B Alzajaji, Abbas A Mansour

**Affiliations:** 1 Faiha Specialized Diabetes, Endocrine and Metabolism Center (FDEMC), College of Medicine, University of Basrah, Basrah, IRQ; 2 Diabetes and Endocrinology, Alhassan Metabolism, Endocrine and Diabetes Center (HMEDC), Karbala, IRQ

**Keywords:** type 2 diabetes, overweight, obesity, smoking, hypertriglyceridemia (htg)

## Abstract

Background

Hypertriglyceridemia (HTG) is one of the major modifiable risk factors for the development of several metabolic diseases. Determining the factors associated with HTG is an important step for increasing awareness of the problem and proper planning of health programs for HTG prevention. This study aimed to determine the conditions associated with HTG in adult patients in Basrah, Iraq.

Methodology

This retrospective study was conducted at Faiha Specialized Diabetes Endocrine and Metabolism Center (FDEMC) in Basra, southern Iraq, in January 2024. The data were retrieved from the center database of 37,133 subjects registered from 2008 to 2023 (16,284, 43.8% males and 20,849, 56.2% females) who attended the FDEMC in Basra due to different reasons.

Results

The most common causes of HTG were type 2 diabetes mellitus (T2DM) (29,799, 80%), obesity (19,914, 53.63%), and smoking (7,309, 12.68%). The age group of 18-45 years displayed higher triglyceride (TG) levels (281.1 ± 210.1 mg/dL) than other age groups. Furthermore, male patients had higher TG levels than females (288.0 ± 196.3 mg/dL vs. 268.9 ± 165.9 mg/dL). Regarding body mass index, overweight and obese patients had higher mean TG levels (284.4 ± 182.1 mg/dL and 281.7 ± 184.6 mg/dL, respectively). Current and ex-smokers had higher TG levels (305.1 ± 212.2 mg/dL and 283.4 ± 161.3 mg/dL, respectively) than non-smokers (272.5 ± 175.4 mg/dL). Moderate HTG was the most common category encountered in 24,137 patients (65%), followed by mild HTG (12,705, 34.2%). Very few patients had severe (264, 7%) or very severe HTG (27, 0.07%). Male patients had more frequent severe and very severe HTG than females.

Conclusions

The most common conditions associated with HTG were T2DM, obesity, and smoking. Smoker males were prone to severe and very severe HTG.

## Introduction

Hypertriglyceridemia (HTG) is typically defined as fasting serum triglyceride (TG) levels of 150 mg/dL or higher. HTG can be caused by a variety of factors, including familial and genetic syndromes, metabolic diseases, and medications. High TG levels are usually seen in conditions such as type 2 diabetes mellitus (T2DM), metabolic syndrome, and familial combined hyperlipidemia. The main cause is an inherited disorder of lipid metabolism, which can be further influenced by secondary causes of dyslipidemia [1]. Different guidelines have specified various cut-off values for categorizing the severity of HTG. The US National Cholesterol Education Program Adult Treatment Panel [2], and the subsequent American Heart Association/American College of Cardiology guidelines on lipid treatment [3], identified serum TG levels of 500 mg/dL or higher as severe HTG, which indicates a risk for pancreatitis. Lower elevations (borderline and borderline high) are associated with an increased risk of atherosclerotic cardiovascular disease (ASCVD). They observed that patients with TG levels in the range of 500-999 mg/dL are at risk of experiencing significant increases in TGs, which could lead to pancreatitis. Pancreatitis is rarely observed when serum TG levels are below 1,000 mg/dL. The Endocrine Society and the European Atherosclerosis Society/European Society of Cardiology define severe HTG as concentrations of at least 1,000 mg/dL [4].

HTG is the most observed form of dyslipidemia in the general population. According to the National Health and Nutrition Examination Survey (NHANES) data [5], around 53% of US adults have dyslipidemia, and 30% have elevated serum TG levels (>150 mg/dL). Encouragingly, there has been a steady decline in the prevalence of HTG [6]. The overall prevalence of HTG, based on NHANES data, is 25.9%. However, in people treated with statins, the prevalence is slightly higher at 31.6% [7]. It is worth noting that the overall prevalence is higher in men (28.7%) than in women (21.5%). Additionally, the highest prevalence is observed in the 40-59-year age group for men and in the over 60-year age group for women [8].

TG levels are lower in children, with levels of 100 mg/dL (1.1 mmol/L) or higher considered abnormal in those aged 0-9 years, and levels of 130 mg/dL or higher deemed abnormal in those aged 10-19 years [3]. Mexican Americans have a nearly double prevalence of high TG levels compared to non-Hispanic Black individuals (34.9% vs. 15.6%). When considering individuals with serum TG levels above 500 mg/dL, the estimated overall prevalence of this level of HTG is 1.7% [9].

Primary genetic dyslipidemia cases are rare. Therefore, primary care focuses on secondary sources such as medications, endocrine conditions, and lifestyle. Primary severe high TGs (HTG) are influenced by both genetic factors that are caused by a single gene (monogenic) and genetic factors that are influenced by multiple genes (polygenic).

A small number of these patients, possibly around 2% of individuals with severe HTG, have monogenic chylomicronemia or familial chylomicronemia syndrome (FCS, formerly known as type 1). This is a rare form of monogenic HTG with an estimated prevalence of 1 to 10 in a million people. The definitive diagnosis of this autosomal recessive disorder requires molecular detection of rare, biallelic variants in one of the genes [10].

The majority of remaining cases of severe HTG are polygenic, which includes contributions from rare heterozygous variants in the canonical *FCS* genes mentioned above and/or common variants associated with elevated TG levels identified in genome-wide association studies [5]. Sometimes, it is called multifactorial HTG (formerly type 5), and certain secondary factors, such as diabetes mellitus, alcohol, estrogen treatment, obesity, and high-fat diets, can worsen the impact of genes on significantly high TG levels [10].

As people age, they may tend to develop excess weight around their midsection, leading to an increase in waist circumference. In combination with a sedentary lifestyle, this can cause insulin resistance and metabolic syndrome. Studies have linked metabolic syndrome to a state that promotes blood clotting and inflammation, which can contribute to endothelial dysfunction and an increased risk of ASCVD [11].

Hormonal imbalances and fluctuations can lead to secondary HTG. Estrogen can significantly stimulate the liver’s production of TGs [12]. During pregnancy, estrogen levels and hence triglyceride levels increase, especially in the third trimester, these levels can rise by up to 200% or more compared to the levels before pregnancy. For women with an underlying genetic disorder of lipid metabolism, this increase in TG levels due to estrogen can increase the risk of pancreatitis and potential fetal loss [11].

Additionally, the intake of oral estrogen through hormone replacement therapy or oral contraceptives may lead to an increase in TG levels due to the same underlying mechanism. However, this effect can be avoided by using topical transdermal or vaginal estrogen because these forms of treatment do not have the same effect because of their reduced exposure to the liver. Tamoxifen, classified as a selective estrogen receptor modulator (SERM), is predominantly used to inhibit the proliferation of estrogen receptor-positive breast cancer cells. Regrettably, one of its side effects is the potential to elevate TG levels. In comparison, raloxifene, another SERM, appears to have a less profound effect on this mechanism [13].

Deficiencies in thyroid hormone can have a significant impact on lipid metabolism. This occurs because of the reduced clearance of TG-rich lipoproteins, which can lead to imbalances in lipid levels and potentially contribute to metabolic disorders [14]. It is recommended to test for thyroid function and screen for hypothyroidism in patients with elevated TG levels during patient workup. Elevated TG levels exceeding 200 mg/dL are commonly found in about 45% of patients with chronic kidney disease owing to the inefficient removal of these particles from the bloodstream. It is crucial to take this into account during clinical evaluation [15].

With alcohol consumption, the liver produces more TGs and TG-rich lipoproteins. This can significantly raise TG levels, especially if combined with other factors. The impact of alcohol on TG levels depends on the amount consumed. When alcohol is consumed with a high-fat meal, as well as in conjunction with conditions such as metabolic syndrome and T2DM, the risk of elevated TG levels becomes even greater [13].

Certain medications can significantly affect plasma TG levels. The most common medications known to cause elevation in TG levels are diuretics, oral estrogens, beta-blockers, immunosuppressants, glucocorticoids, antipsychotic medications, antiretroviral therapy isotretinoin, and bile acid sequestrants [11].

In patients with high TG levels, it is important to re-evaluate the use of antihypertensive medications such as thiazide diuretics, furosemide, and beta-adrenergic blockers. Atenolol, metoprolol, and propranolol have a more pronounced effect on TG levels compared to carvedilol. In addition, antipsychotic medications such as clozapine, risperidone, and quetiapine may cause HTG and metabolic syndrome. This effect has not been seen with aripiprazole and ziprasidone [13].

## Materials and methods

Study design, place, and duration

This retrospective study was conducted at Faiha Specialized Diabetes Endocrine and Metabolism Center (FDEMC) in Basrah, southern Iraq, in January 2024. The data were retrieved from the center database of 37,133 subjects registered from 2008 through 2023 (16,284, 43.8% males and 20,849, 56.2% females) who attended the center for different disorders. Informed consent was obtained from all participants and the study was approved by the FDEMC Ethical Committee (approval number: 56/35/22).

Inclusion and exclusion criteria

**Figure 1:**
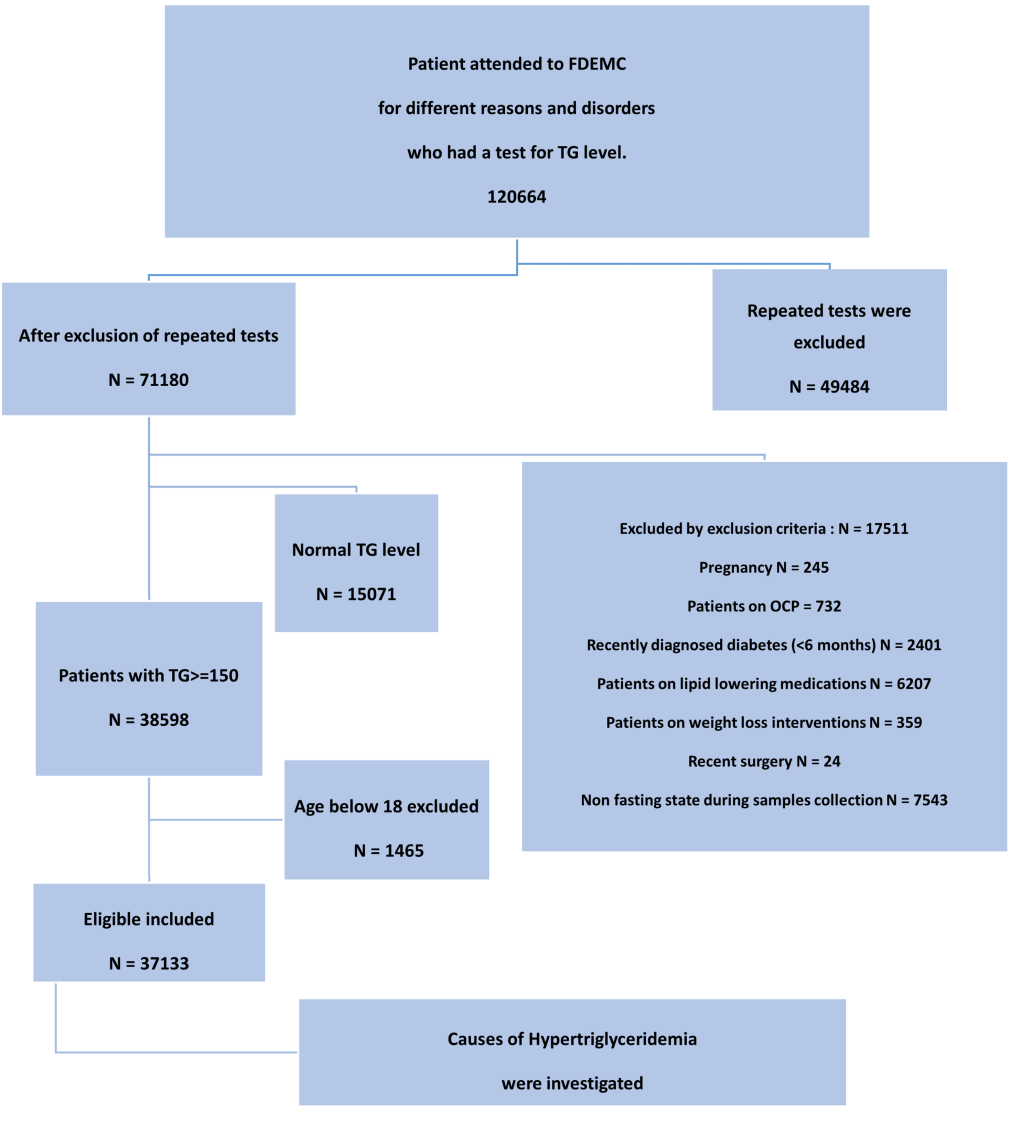
Study design. FDEMC = Faiha Specialized Diabetes Endocrine and Metabolism Center; TG = triglyceride; OCP = oral contraceptive pills

We included patients aged 18 years who visited FDEMC for various reasons including screening. We excluded pregnant patients, those recently diagnosed with diabetes (within six months), those below 18 years of age, those on lipid-lowering medications, those on weight loss interventions, those who had recently undergone surgery, those in a non-fasting state during sample collection, and patients on oral contraceptive pills (Figure 1).

Data collection

We gathered clinical and biochemical information, took precise measurements of weight and height, and then computed the body mass index (BMI). A detailed lipid profile was also reported. All data were collected from center data. All biochemical assays were conducted by a consistent team of laboratory technicians utilizing the enzymatic assay (glycerol-phosphate-oxidase-peroxidase-amidopyrine method) using the Hitachi Roche Cobas C311 machine for biochemistry. Hemoglobin A1c (HbA1c) was checked using the Bio-Rad variant II machine. The Endocrine Society reference range and classification for HTG severity were used. Mild HTG was defined as TG levels of 150-199 mg/dL, moderate HTG as TG levels of 200-999 mg/dL, severe HTG as TG levels of 1,000-1,999 mg/dL), and very severe HTG as TG levels >2,000 mg/dL.

Statistical analysis

The data were described using descriptive statistics such as mean, standard deviation, frequency, and percentage. For continuous variables, central tendencies were expressed as means and standard deviation, and for categorical variables, they were expressed as percentages. All statistical analyses were conducted using SPSS Statistics version 25.0 (IBM Corp., Armonk, NY, USA), with a p-value <0.05 considered statistically significant.

## Results

The mean age of the patients was 50.2 ± 12.2 years. The age group of 45-65 years was the most common, accounting for 56.8% of the patients, followed by the age group of 18-45 years (31.1%). Females were more common than males (56.1% vs. 43.9%). Normal weight and overweight patients were the most common BMI categories, accounting for 31.6% and 31%, respectively. Only 14.6% and 8% were obese or morbidly obese, respectively. Hypertension was reported in 41.1% of the patients. Most of the patients (81.5%) were non-smokers. However, 12.7% and 5.8% were either current or ex-smokers, respectively. The mean serum total cholesterol (TC), HDL, non-HDL, LDL, and TG were 203.7 ± 50.2 mg/dL, 40.7 ± 13.4 mg/dL, 161.1 ± 50.2 mg/dL, 127.5 ± 43.0 mg/dL, and 277.3 ± 180.1 mg/dL, respectively (Table 1).

**Table 1:** Demographic characteristics of the patients. *: 750 patients have no BMI measurements due to different causes such as being wheelchair-bound. BMI = body mass index; SBP = systolic blood pressure; DBP = diastolic blood pressure; TC = total cholesterol; HDL-C = high-density lipoprotein cholesterol; NHDL-C = non-high-density lipoprotein cholesterol; LDL-C = low-density lipoprotein cholesterol; TG = triglycerides

Variable	
Age (years), mean ± SD	50.2 ± 12.2
18–45, N (%)	11,547 (31.1%)
45–65, N (%)	21,081 (56.8%)
65+, N (%)	4,505 (12.1%)
Male, N (%)	16,284 (43.9%)
Female, N (%)	20,849 (56.1%)
BMI (kg/m^2^)*, mean ± SD	31.3 ± 6.1
<18.5, N (%)	246 (0.7%)
18.5–25, N (%)	4,478 (12.1%)
25–30, N (%)	11,746 (31.6%)
30–35, N (%)	11,523 (31.0%)
35–40, N (%)	5,427 (14.6%)
40+, N (%)	2,964 (8.0%)
Smokers, N (%)	4,709 (12.7%)
Ex-smoker, N (%)	2,165 (5.8%)
Non-smoker, N (%)	30,259 (81.5%)
Hypertensive, N (%)	15,274 (41.1%)
Non-hypertensive, N (%)	21,859 (58.9%)
SBP (mmHg), mean ± SD	138.3 ± 21.8
DBP mmHg), mean ± SD	83.6 ± 12.3
TC (mg/dL), mean ± SD	203.7 ± 50.2
HDL-C (mg/dL), mean ± SD	40.7 ± 13.4
NHDL-C(mg/dL), mean ± SD	161.1 ± 50.2
LDL-C (mg/dL), mean ± SD	127.5 ± 43.0
TG (mg/dL), mean ± SD	277.3 ± 180.1

**Figure 2:**
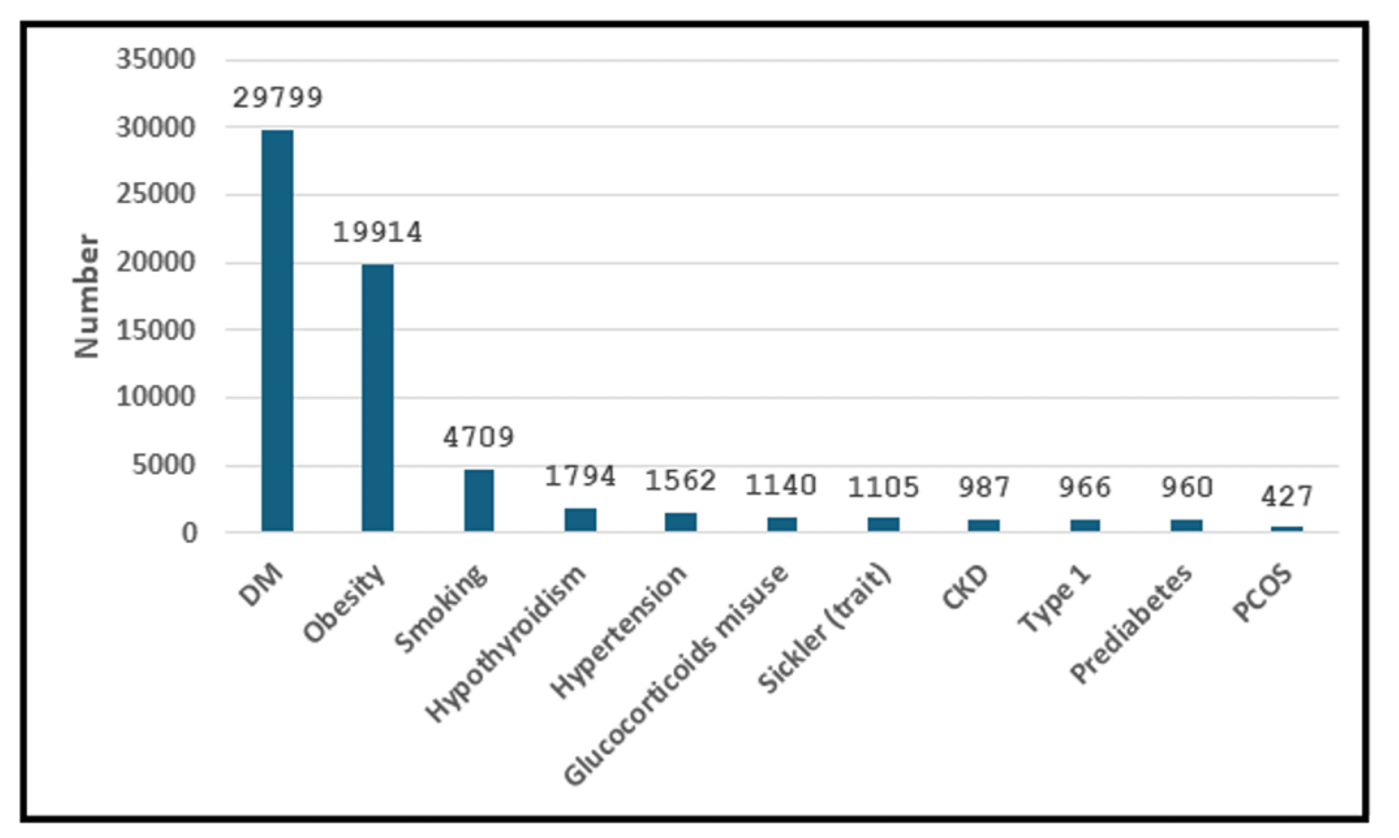
Common associations of hypertriglyceridemia. CKD = chronic kidney disease; DM = diabetes mellitus; PCOS = polycystic ovary syndrome

The most common association of HTG were T2DM (80%), obesity (53.63%), and smoking (12.68%). Less common include hypothyroidism, hypertension, glucocorticoid misuse, sickle cell anemia, chronic kidney disease, type 1 DM, prediabetes, and polycystic ovary syndrome (4.83%, 4.21%, 3.07%, 2.98%, 2.66%, 2.6%, 2.59%, and 1.15%, respectively) as shown in Figure 2.

The age group of 18-45 years displayed higher TG levels (281.1 ± 210.1 mg/dL) than other age groups. Furthermore, male patients had higher TG levels than females (288.0 ± 196.3 mg/dL vs. 268.9 ± 165.9 mg/dL). Regarding BMI, overweight and obese patients had higher mean TG levels (284.4 ± 182.1 mg/dL and 281.7 ± 184.6 mg/dL, respectively). Hypertension seems to have no significant impact on TG levels. In contrast, current and ex-smokers had higher TG levels (305.1 ± 212.2 mg/dL and 283.4 ± 161.3 mg/dL, respectively) than non-smokers (272.5 ± 175.4 mg/dL) (Table 2).

**Table 2:** Factors found to affect TG levels. BMI = body mass index; TG = triglycerides

Factor	TG (mg/dL) N ± SD	P-value	
Age (years)	TG (mg/dL) N ± SD		
		<0.001	
18–45	281.1 ± 210.1		
45–65	278.9 ± 170.7		
65+	259.7 ± 123.4		
Sex	TG (mg/dL) N ± SD		
Male	288.0 ± 196.3	<0.001	
Female	268.9 ± 165.9		
BMI (kg/m^2^)	TG (mg/dL) N ± SD		
<18.5	257.5 ± 202.0	<0.001	
18.5–25	284.4 ± 182.1		
25–30	281.7 ± 184.6		
30–35	273.3 ± 146.7		
40+	259.1 ± 190.9		
Smoking	TG (mg/dL) N ± SD		
Non-smoker	272.5 ± 175.4	<0.001	
Smoker	305.1 ± 212.2		
Ex-smoker	283.4 ± 161.3		
Hypertension	TG (mg/dL) N ± SD		
Yes	279.1 ± 168.9	0.098	
No	276.0 ± 187.5		

**Figure 3:**
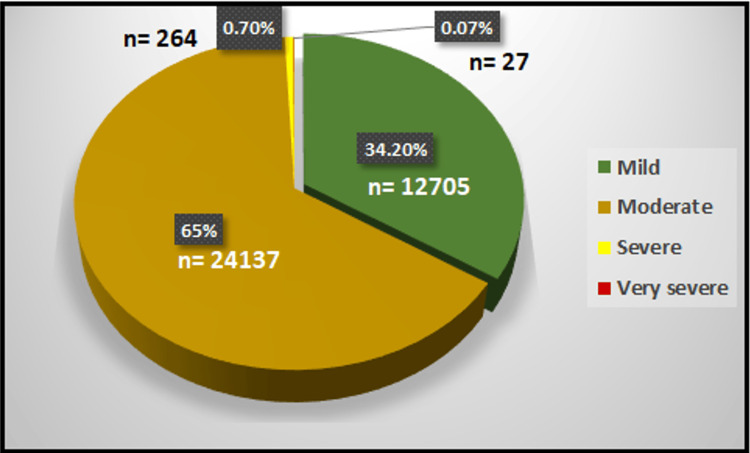
Severity of hypertriglyceridemia.

Moderate HTG was the most common category encountered in 24,137 patients (65%), followed by mild HTG in 12,705 patients (34.2%). Very few patients had severe HTG (264 patients, 0.7%) or very severe HTG (27 patients, 0.07%), as shown in Figure 3.

Generally, the average age of patients with severe and very severe HTG was lower than that of those with mild and moderate HTG. Moreover, male patients had more frequent severe and very severe HTG than females. Additionally, current smokers were more common in severe and very severe HTG than ex-smokers and non-smokers, as shown in Table 3.

**Table 3:** Characteristics of patients in different TG severity levels. BMI = body mass index; HTG = hypertriglyceridemia; TG = triglycerides

Factor	Mild HTG	Moderate HTG	Severe HTG	Very severe HTG	P-value
Age (mean ± SD) (years)	49.9 ± 13.0	50.5 ± 11.8	45.8 ± 11.3	42.1 ± 12.7	<0.001
18–45	4,202 (36.4)	7,209 (62.4)	119 (1.0)	17 (0.1)	<0.001
45–65	6,837 (32.4)	14,107 (66.9)	129 (0.6)	8 (0.04)	
65+	1,666 (37)	2,821 (62.6)	16 (0.4)	2 (0.04)	
Sex					
Male, N (%)	5,256 (32.3)	10,852 (66.6)	158 (1.0)	18 (0.1)	<0.01
Female, N (%)	7,449 (35.7)	13,285 (63.7)	106 (0.5)	9 (0.04)	
BMI (kg/m^2^), mean ± SD	31.2 ± 6.5	31.3 ± 5.9	30.2 ± 5.2	30.2 ± 5.5	0.022
Obese, N (%)	6,690 (33.6)	13,086 (65.7)	127 (0.6)	11 (0.1)	0.003
Non obese, N (%)	5,739 (34.8)	10,579 (64.2)	136 (0.8)	16 (0.1)	
Smoking					
Smoker, N (%)	1,401 (29.8)	3,232 (68.6)	67 (1.4)	9 (0.2)	<0.001
Ex-smoker, N (%)	679 (31.4)	1,468 (67.8)	18 (0.8)	1 (0.05)	
Non-smoker, N (%)	10,625 (35.1)	19,437 (64.2)	180 (0.6)	17 (0.1)	
Hypertension					
Yes, N (%)	4,973 (32.6)	10,185 (66.7)	112 (0.7)	4 (0.03)	<0.001
No, N (%)	7,732 (35.4)	13,952 (63.8)	152 (0.7)	23 (0.1)	
SBP, mean ± SD (mmHg)	137.4 ± 22.0	139.7 ± 21.6	137.9 ± 20.9	136.5 ± 22.5	<0.001
DBP, mean ± SD (mmHg)	82.7 ± 12.4	84.1 ± 12.2	85.5 ± 13.2	83.1 ± 11.1	<0.001

## Discussion

In this study, most patients with HTG had T2DM, with around half being obese and a minority being smokers. Alfhaid [15] recruited 353 Saudi individuals and reported an HTG prevalence of 33.7%. The most common causes were T2DM and metabolic syndrome (55.5% and 34.6%, respectively). Abujbara et al. [16] conducted a study in Jordan investigating a group of 4,056 individuals ranging in age from 18 to 90 years. The prevalence rate in the study was 41.9%. In this investigation, a strong correlation was noted between obesity, smoking, and T2DM and the occurrence of HTG.

A study conducted in Iran by Khanali et al. among 18,119 persons aged 25 years and older found that the prevalence rate of HTG was 39.7% [17]. Hypertension and obesity were identified as factors related to HTG. However, the occurrence of HTG was not affected by smoking or T2DM. Ford et al. [18] demonstrated that advanced age, male gender, current smoking, alcohol consumption within the last year, being overweight or obese, and having diabetes were all strongly linked to high TG levels in the American population.

Lipid abnormalities are frequently observed in individuals with T2DM, and these abnormalities contribute considerably to the elevated cardiovascular risk in T2DM patients [19]. Patients with T2DM often experience elevated TG levels, even when their T2DM is well controlled. This is likely due to the presence of additional conditions such as obesity, smoking, alcohol consumption, or the apo E2/E2 genotype [20].

The mechanism of HTG in obesity involves insulin resistance, which results in an elevated flow of free fatty acids (FFA) from belly fat to the liver. This leads to increased production of very low-density lipoprotein (VLDL) in the liver. HTG is further exacerbated due to the struggle for clearance between VLDL and chylomicrons, as both undergo the same breakdown mechanisms. Nevertheless, it is important to note that not all individuals who are overweight will develop HTG. Several studies have demonstrated that elevated levels of plasma apo C-III, which hinder the breakdown of TGs, play a substantial role in the development of hypertriglyceridemic waist [21]. Smokers exhibited an elevation in chylomicrons and their corresponding residues after eating, which was marked by an increase in postprandial apo B48 concentrations. The elevated quantity of chylomicrons and chylomicron remnants is likely a result of reduced elimination of chylomicron remnants [22].

In this study, around two-thirds of patients had moderate HTG, around one-third had mild HTG, and very had severe and very severe HTG. Age, BMI, and smoking were significantly associated with the severity of HTG. The primary factors linked with extremely severe HTG were uncontrolled diabetes mellitus, with secondary associations with heavy alcohol consumption, medication use, and hypothyroidism. A study from Denmark, involving 108,711 participants from the Copenhagen General Population Study, yielded findings consistent with our own. This study highlighted T2DM and obesity as the most noteworthy risk factors [23].

According to Patni et al. [24], the primary factor associated with extremely severe HTG in children is uncontrolled diabetes (31%), followed by the use of L-asparaginase and high-dose steroids for children with acute lymphoblastic leukemia (28%). A study conducted in Japan by Tada et al. [25] among 5,251 individuals with TG levels exceeding 1,000 mg/dL revealed that most patients (59%) developed HTG because of both diabetes and excessive alcohol consumption. Overall, 27% of the cases were attributed to a monogenic etiology.

A Spanish study involved a total of 428,334 males and 166,367 females, with an average age of 36 ± 10 years. The study revealed that 95,673 individuals, which accounted for 16% of the participants, had mild HTG. Of those, 1.1% had moderate HTG, while 0.03% had severe HTG. Among individuals diagnosed with HTG, 90% were males. HTG was found to be related to age, obesity, T2DM, alcohol intake, and vascular disease. Obesity was found to be the main independent predictor of mild HTG, with an odds ratio (OR) of 2.42 and a 95% confidence interval (CI) of 2.37-2.48. On the other hand, diabetes was identified as a predictor of moderate HTG, with an OR of 3.64 and a 95% CI of 3.17-4.18, as well as severe HTG, with an OR of 7.35 and a 95% CI of 4.27-12.66 [26].

In a recent case-control study, diabetes was found to be the primary factor associated with severe HTG, even after considering genetic predispositions. In their analysis of data from 5,680 individuals aged over 20 years, Christian et al. [8] determined that approximately 1.7% of the study sample exhibited severe HTG. This study was part of the National Health and Nutrition Examination Survey and aimed to assess the epidemiology of severe HTG in adults. Most subjects with severe HTG were male (75.3%) and between the ages of 40 and 59 years (58.5%). As much as 14% of individuals with severe HTG had T2DM, whereas 31.3% were suffering from hypertension. In addition, the researchers discovered that severe HTG was significantly lower in women compared to men (25% vs. 75%).

The study has a few limitations. As it is a retrospective study, a cause-effect relationship between HTG and other factors could not be determined. The information regarding the diet of the included participants was not obtained. It was not possible to gather information regarding genetic studies.

## Conclusions

The most common conditions associated with HTG were T2DM, obesity, and smoking. Overweight/obese smokers, especially younger age, are more prone to HTG, and the middle-aged group displayed higher TG levels, with the vast majority of patients having mild or moderate HTG. Current smoker males can develop severe and very severe HTG if they ignore proper protective measures. Thus, all efforts to reduce HTG should focus on modifiable factors, especially smoking and obesity.
